# First case of huge classic papillary thyroid cancer rupturing spontaneously leading to ischemic necrosis, perforation and inflammation of overlying skin: Case report and review of the literature

**DOI:** 10.1016/j.ijscr.2021.106136

**Published:** 2021-06-29

**Authors:** Abdelrahman Abusabeib, Walid El Ansari, Mohamed S. Al Hassan, Mahir Petkar, Sugad Mohamed

**Affiliations:** a Department of General Surgery, Hamad General Hospital, Hamad Medical Corporation, Doha, Qatar; b Department of Surgery, Hamad General Hospital, Hamad Medical Corporation, Doha, Qatar; c College of Medicine, Qatar University, Doha, Qatar; d School of Health and Education, University of Skovde, Skovde, Sweden; e Department of Laboratory Medicine & Pathology, Hamad Medical Corporation, Doha, Qatar

**Keywords:** Thyroid cancer, Papillary thyroid cancer, Lymphocytic thyroiditis, Ischemic necrosis, Case report

## Abstract

**Introduction:**

Papillary thyroid cancer (PTC) is the commonest form of well-differentiated endocrine carcinoma. It is categorized into indolent and aggressive, where the indolent subtypes (classic, follicular) rarely demonstrate aggressive behavior. We present a classic PTC presenting with a rapidly growing huge anterior neck mass that subsequently spontaneously ruptured subcutaneously resulting in ischemia, necrosis, and perforation of overlying skin leading to inflammation.

**Presentation of case:**

A 37-year-old female with no comorbidities presented to our emergency department with a neck swelling of 2 years duration that rapidly enlarged one week prior to presentation. Though the mass initially appeared of inflammatory nature, the tumor was a PTC, and she underwent total thyroidectomy with selective right side neck dissection and debridement of necrotic skin. The gross specimen revealed a fragmented non-intact right thyroid lobe mass causing pressure ischemia, necrosis and perforation of the skin. Histopathology showed a 9 × 9 × 5 cm classic PTC staged as pT3b N1b. Postoperative course was uneventful, she was discharged by the eighth postoperative day, and then she received a high dose of radioactive iodine ablation (RAI).

**Discussion:**

Classic PTC is usually of a smaller size and a relatively benign course compared to other PTC subtypes and thyroid cancers. It is indolent with favorable prognosis. Although it is associated with increased risk of lymph node metastases at the time of diagnosis, it is slow growing with high survival rates approaching 95%.

**Conclusion:**

Despite that classic PTC progresses slowly, it should still be suspected in neck swellings presenting with rapid and aggressive behavior. Prompt and systematic assessment is required with surgical intervention and radioactive iodine ablation therapy.

## Highlights

• Classic PTC is of indolent nature, slow progression and favorable prognostic outcomes when treated.
• We present a huge mass, rapid progression, spontaneous rupture causing ischemia, necrosis and perforation of overlying skin.
• Systematic investigations are required using US, FNA and CT followed by surgical intervention is the mainstay of treatment.
• High risk patients must undergo RAI soon after surgery with semiannual then annual follow-up to assess any recurrence.

## 1 Background

Papillary thyroid cancer (PTC) is the most common form of thyroid cancer, globally increasing in incidence with a presumption to soon become the third most common cancer in females [1], [2]. Though increasing in incidence, PTC demonstrates favorable prognosis, with more than 93% ten-year survival rate [3]. PTC mainly manifests as a neck mass and thyroid nodule, with common locoregional metastasis to the surrounding lymph nodes of the neck, but rarely with distant metastasis [4], [5]. Recently, new histopathologic variants of PTC have been classified into indolent (classical, follicular, macrofollicular) and aggressive (hobnail, tall cell, columnar cell, clear cell, solid, diffuse sclerosing) categories [6].

In terms of size, PTC is classified into microcarcinoma (≤1.5 cm); intrathyroidal (>1.5 cm); and the extrathyroidal type that normally breaches the thyroid capsule into the surrounding structures [7]. Size alone (>2 cm) has more aggressive biological characteristic and is an independent factor for local and distant recurrences [8]. One PTC (hobnail variant) reached 14 cm [9]; and others reported large PTC tumors that mostly consisted of cystic components [10], [11], [12].

PTC is frequently associated with chronic lymphocytic thyroiditis (CLT), the most common inflammatory disorder of the thyroid gland with an incidence of 0.5–38% [13]. Prognosis of PTC remains outstanding with treatment, typically comprising surgery followed by radioactive iodine (RAI) ablation, which is mostly curative for the majority of PTCs [14].

We report a classic PTC presenting as a huge anterior neck mass (9 cm long axis) that subsequently spontaneously ruptured subcutaneously resulting in ischemia, necrosis, and perforation of overlying skin and leading to inflammation of the anterior region of the neck. To the best of our knowledge, this is the first report of a large sized, predominantly solid, classic PTC that subsequently spontaneously ruptured subcutaneously resulting in ischemia, necrosis, perforation of the overlying skin and secondary inflammation. No previous published reports have described such a case. We report this case in line with the updated consensus-based surgical case report (SCARE) guidelines [15]. In addition, we also conducted a literature review of large fast-growing PTCs.

## 2 Case presentation

A 37-year-old Indonesian female presented to the emergency department (ED) at our institution in late August 2020 with a huge neck swelling that gradually progressed in size over 2 years. In the last 7 days prior to presentation, the swelling rapidly increased in size, became associated with pain and dysphagia that resulted in mainly liquid oral intake, in addition to a dark brown discoloration of the overlying skin. There was no change in voice, and no shortness of breath, and no fever, night sweats or loss of appetite. She had no comorbidities; no history of trauma and the systemic review was unremarkable. The patient is non-smoker, not on any medications, with no relevant past medical or family history.

**Fig. 1:**
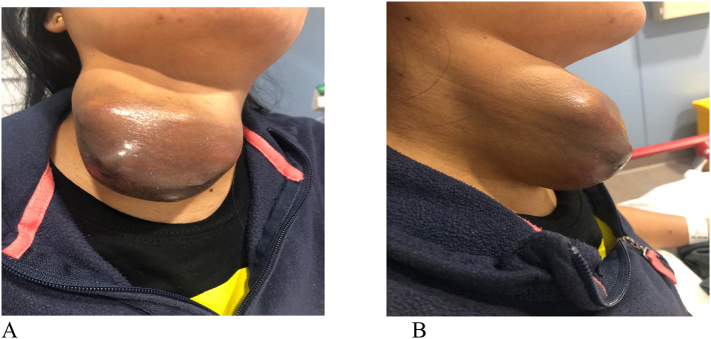
Front (A) and lateral (B) views of huge thyroid mass extending beyond the midline, with shiny ischemic dark brown discolored skin.

Clinical examination revealed a well-built, afebrile and vitally stable female with a massive swelling in the anterio-lateral aspect of her neck extending more to the right side with palpable jugular cervical lymph nodes. The skin overlying the mass was shiny, with an area of dark brown discoloration, patchy necrosis and skin perforation through which thin serous dark brown odorless discharge was noted. The underlying mass was tender, partially fluctuant, and it was difficult to assess its mobility or attachment to underlying structures due to the tenderness and large size (Fig. 1A and B).

## 3 Investigations

### 3.1 Blood

Blood tests revealed normal white blood count, slightly elevated erythrocyte sedimentation rate (ESR) 28 mm/h, C-reactive protein of 7.2 mg/l, and thyroid function (TSH, free T4) within normal range. In terms of tumor markers, anti-thyroglobulin antibodies were within normal limits, while thyroglobulin was elevated (924 ng/ml).

### 3.2 Chest X-ray

Postero-anterior chest X-ray was within normal apart from neck opacity.

### 3.3 Ultrasound (US) of the neck

**Fig. 2:**
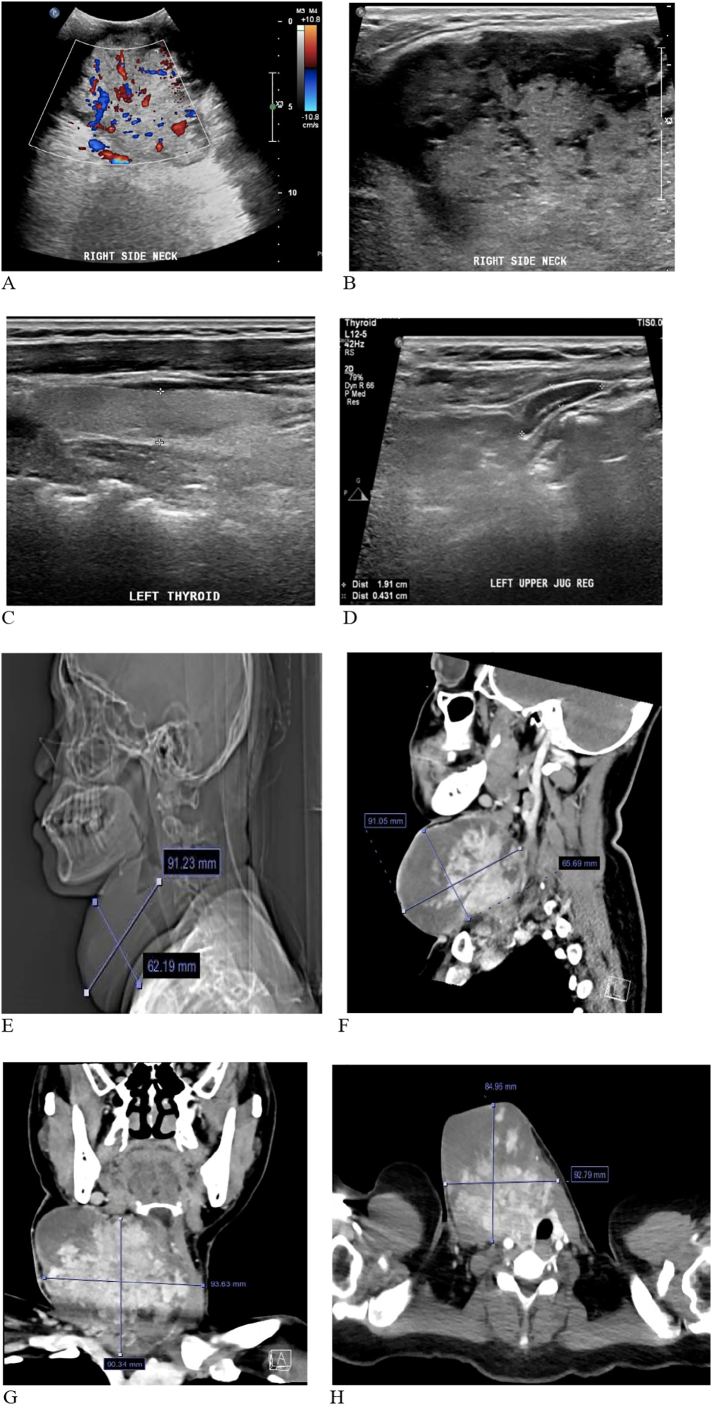
Thyroid imaging. Ultrasound. (A) Right lobe revealing large complex heterogeneous mass (9.4 × 8 cm) with midline extension from which right lobe is inseparable. (B) Doppler evaluation of right lobe showing internal vascularity within the lesion. (C) Unremarkable left lobe. (D) Largest lymph node (19 × 4 mm) in left upper jugular region. Head and chest CT findings. (E) and (F) Plain and contrast sagittal views revealing large right thyroid mass with pressure effect on adjacent structures. (G) and (C) Coronal and transverse views revealing large thyroid mass.

This revealed a large complex heterogeneous mass with internal vascularity noted on doppler evaluation (Fig. 2A), originating from the right side with midline extension, measuring approximately 9.4 × 8 cm, from which the right thyroid lobe could not clearly be separated (Fig. 2B). The left lobe of thyroid appeared unremarkable (Fig. 2C). Multiple cervical lymph nodes were noted, the largest measuring 19 × 4 mm in the left upper jugular region (Fig. 2D). Both parotid and submandibular glands appeared unremarkable.

### 3.4 Computed tomography (CT) scan of the neck and thorax

This revealed a large right thyroid mass with mass effect on adjacent structures, highly suggestive of malignancy (Fig. 2E, F, G, H).

### 3.5 Cytopathology

Ultrasound-guided fine needle aspiration (FNA) for cytology was undertaken with a 23 Gauge needle 10 ml syringe with suction for a total of 2 passes. An aspirate of 1 ml of dark brown fluid was obtained, and microscopic assessment revealed a cellular smear with many papillary follicular epithelial cells, nuclear grooves, foamy macrophages in the background of blood, diagnostic of PTC.

## 4 Surgical technique and findings

The patient was discussed at our thyroid cancer multi-disciplinary team (MDT) meeting and the recommendation was for total thyroidectomy with selective right side neck dissection. As per the US finding of left upper jugular lymph node (19 × 4 mm), it demonstrated benign appearance (preserved shape and hilum). Hence, the MDT recommended selective right side neck dissection only. Prior to surgery, she underwent vocal cords assessment as a routine pre-thyroid surgery evaluation which revealed normal findings. She continued a 5 day antibiotic course for the inflammation to settle. Then the results of her investigations were ready, and she underwent surgery.

**Fig. 3:**
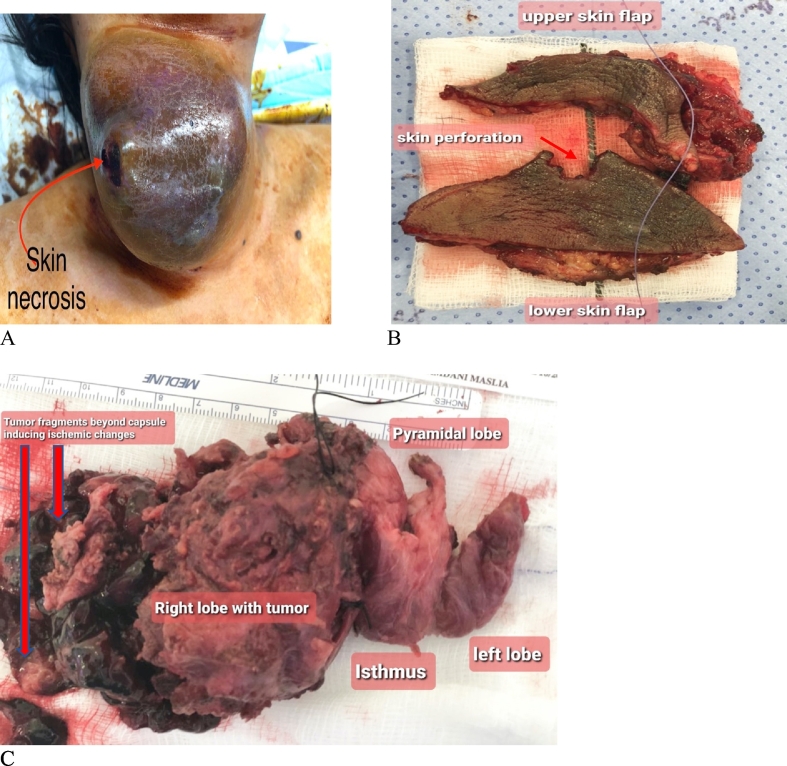
Intraoperative findings. (A) Anterior neck mass prepped with iodine demonstrating skin necrosis with perforation. (B) Upper and lower skin flaps with ischemia, pressure necrosis and perforation of the skin. (C) Gross thyroid specimen with huge right thyroid lobe fragmented mass that ruptured beyond the capsule.

At our facility, the patient was booked and prepared for urgent surgery. The patient underwent the standard preparation for the procedure which was undertaken by experienced consultants in thyroid surgery and anesthesia. She was intubated (orotracheal) under aseptic measures, and then placed in appropriate thyroid surgery position (supine position, 15 degrees head elevation, neck extension over ring support along with shoulder support via bean bag, both hands tugged to the body). She was prepped with iodine and draped (Fig. 3A), and an approximately 5 × 10 cm elliptical incision on the anterior aspect of the neck was undertaken including all the necrotic perforated skin patch (Fig. 3B).

**Fig. 4:**
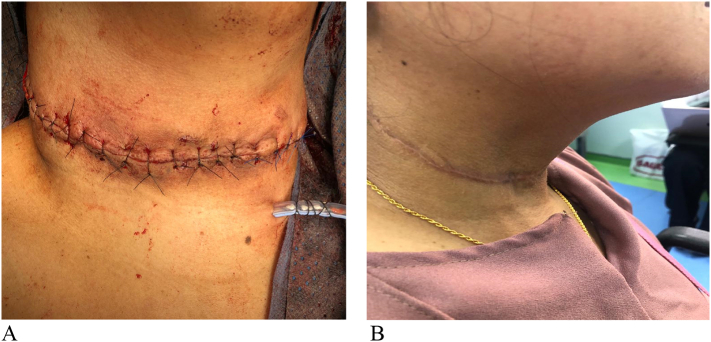
Skin incision and scar. (A) Immediate post-surgery, showing skin closure with ethilon in simple interrupted fashion with drain. (B) Six-month postoperative follow up showing optimal wound healing.

Total thyroidectomy with selective right side neck dissection was undertaken. Intraoperatively, the tumor was found to be partially fragmented (not completely intact, denoting spontaneous rupture). It extended beyond the thyroid capsule hence generating a mass effect with ischemia to the surrounding and subcutaneous tissues and necrosis of the overlying skin which eventually led to the perforation of the skin (Fig. 3B, 3C). The tumor was also adhered to the right strap muscles for which partial excision was done. The recurrent laryngeal nerves and parathyroid glands were preserved bilaterally, and the central together with level 2–5 lymph nodes on the right side where selectively dissected. The specimen was marked using silk sutures and fixed in a formalin container. A surgical drain was placed followed by closure in layers and the skin was closed in simple interrupted fashion using Ethilon 4/0 suture (Fig. 4A) with the intention to detect and intervene in any early postoperative complication (hematoma). The patient was smoothly extubated in the operating room and escorted to the surgical intensive care unit for 24 h observation as recommended by the anesthetist. She was transferred to the ward after 24 h, the postoperative course was uneventful and the patient was discharged by the eighth postoperative day.

## 5 Pathology

**Fig. 5:**
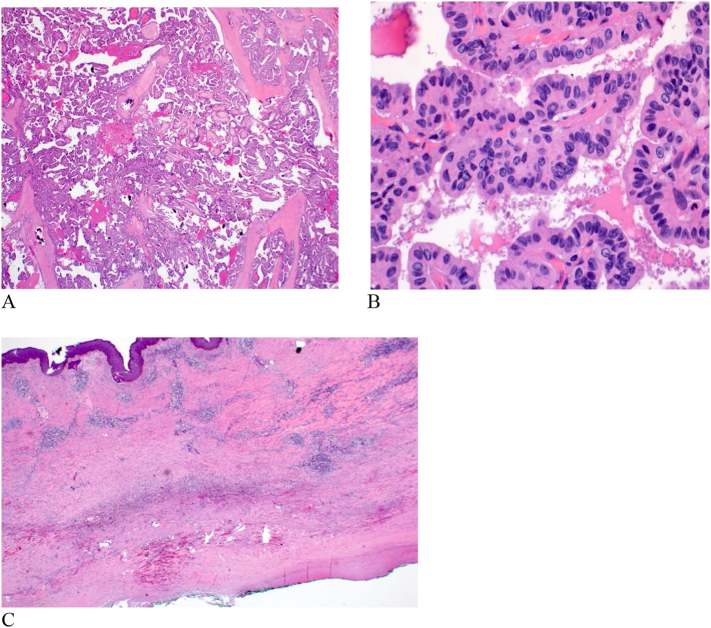
Histopathology of thyroid and skin. (A) Low power showing classical papillary architecture of PTC (H and E x 2). (B) Tumor cells displaying characteristic nuclear features, nuclear overlapping, grooves and pseudoinclusions (H and E x 20). (C) Skin showing marked inflammation with areas of hemorrhage and congestion, in keeping with ischemic changes (H and E x2).

Histopathology examination of the specimen revealed a unifocal right thyroid lobe classic PTC, measuring 9 × 9 × 8 cm (maximum dimension). Microscopically, the lesion exhibited papillary architecture, with nuclear overlapping, nuclear grooves and pseudo-inclusions. The carcinoma extended to the resection margins. Background CLT was noted. Sections from the overlying skin displayed marked mixed inflammation and congestion, consistent with early ischemic changes (Fig. 5A, B, C). In addition, 4 out of 24 cervical lymph nodes were positive for metastatic PTC and the tumor was thus staged as pT3b N1b (pTNM, AJCC 8th Edition).

## 6 Follow-up

**Fig. 6:**
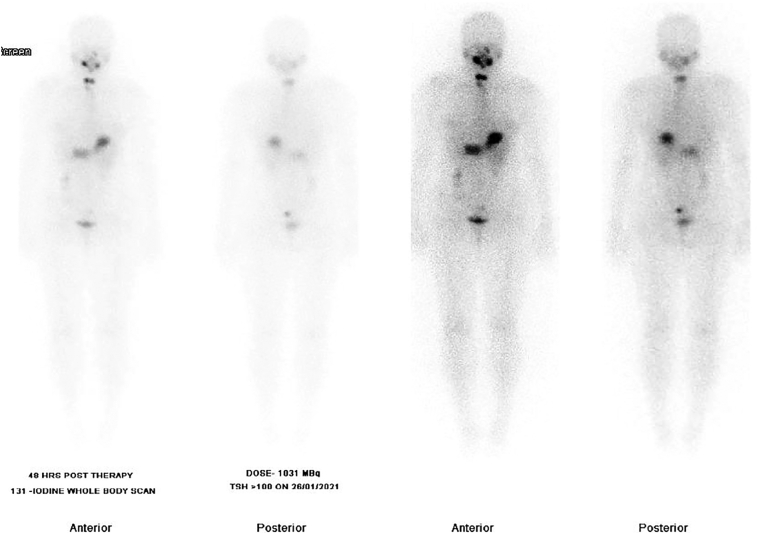
Nuclear medicine (NM) 131 iodine scan revealing focal right and left thyroid bed region uptake.

The post-operative period was uneventful. In the initial period after the surgery, the wound showed optimal healing with no immediate or late complications. She was started on daily 75 μg of thyroxine upon discharge on the 8th postoperative day. The case was re-discussed at the thyroid surgery MDT meeting, and given the histopathology findings and staging, she was recommended for high dose radioactive iodine ablation (RAI) therapy, for which she received Thyrogen injection followed by administration of oral 1031 MBq iodine-131. She then undertook nuclear medicine (NM) 131 iodine scan which revealed focal right and left thyroid bed region uptake (Fig. 6), with successful delivery of I-131 to the mentioned foci. The patient was followed for 6 months with optimum wound healing (Fig. 4B) and no signs of local recurrence or complications noted.

## 7 Discussion

Recently, new relatively more aggressive PTC subtypes have been reported (e.g., hobnail, tall cell, columnar cell, clear cell, solid, diffuse sclerosing). However, the most common variant (50%) remains the well differentiated (classic, follicular, conventional) subtype. This subtype exhibits a smaller tumor size, relatively favorable course, slow progression, indolent features of <1% extra nodal metastasis and 28% nodal metastasis on presentation, and high survival rate [6], [16], [17]. Four cases have been previously published of locally huge masses with rapid expansion. These comprised one hobnail PTC [9] and three cystic PTCs [10], [11], [12].

One of these cystic PTCs [12] demonstrated resemblance to the current case in terms of its rapid growth (15 days) and pain. However, the general condition of this case [12] pointed to an infectious process, evident by the cellulitis, fever (38C), purulent discharge, leukocytosis (13,500 10^9^/ul), high CRP (42 mg/dl), and the FNA showed purulent material without cytologic signs of malignancy. In addition, the tumor (11 cm) was mostly cystic, invading the surrounding structures (aggressive tumor unlikely to be of the classical type) [12]. All these characteristics were not represented in our case. Contrary to this clinical picture, our patient had a large (9 × 9 × 8 cm) classic PTC presenting as a gradually increasing mass over 2 years followed by rapid growth for 7 days prior to presentation. This rapid growth was due to the spontaneous subcutaneous rupture of the tumor resulting in a significant increase in size, along with pain and skin discoloration due to the pressure ischemic effect and the inflammatory process eventually developing a patch of skin necrosis and perforation. In addition, our patient was afebrile, had serous non-purulent discharge, no leukocytosis, only slightly elevated CRP (7.2 mg/l), and the FNA showed dark brown fluid with cellular smear and many papillary follicular epithelial cells, nuclear grooves, foamy macrophages in the background of blood, diagnostic of PTC. Furthermore, our case had only local lymph nodes metastasis (4 out of 24 cervical lymph nodes were positive for metastatic PTC), with no evidence of invasion to the surrounding structures. To the best of our knowledge, this is the first published report of such a case.

In terms of age and gender, PTC tends to occur more frequently among younger individuals, with a peak between 30-50 years, and is more common in females (female-to-male ratio 3.6:1) [18]. The literature review we undertook of PTC subtypes presenting as fast-growing huge masses (Table 1) is in agreement, as all the identified cases were females [9], [10], [11], [12], and almost all were within the above age distribution, with the exception of one report of a 60 years old female [11]. As for presentation, PTC is usually asymptomatic except for locally advanced cases that might present with pressure symptoms (e.g., dysphagia, dyspnea, change in voice) and palpable lymph nodes [19]. Our patient agrees with such description, presenting with a rapidly growing anterior neck swelling causing dysphagia mainly to solids but no other pressure symptoms. However, the patient also complained of pain that she developed one week before she sought medical advice, which is a rather unusual presentation for PTC. Unlike the current case where the PTC was mostly solid lesion, Table 1 identified other PTC cases with large growth of which three were mainly large cystic swellings but none reported pressure symptoms or pain [10], [11], [12]. However, one published report was of a similar tumor component like ours (mostly solid) but of a different variant of PTC presenting with a 3-month thyroid swelling, accompanied with dysphagia, odynophagia and slight change in voice, but no pain [9]. Our review identified another case of anaplastic thyroid cancer which also displayed rapid growth in an even shorter duration (4 months), but was not included in Table 1 as it is another type of aggressive thyroid cancer [20].

**Table 1:** Literature review: comparison of current case with other PTC subtypes presenting as fast-growing huge mass.

Case	A/G	D	S	CP	Blood	Radiology	FNAC	T	HP	EE	LN/+	TNM	RAI	Re/f
Current case	F 37	28	9 × 9 × 8	Massive mass, anterior-lateral aspect of neck, extend to right side, skin overlying shiny, erythematous, blanching under pressure, with blackish discoloration	High: CRP 9.3 mg/l; ESR 28 mm/h; thyroglobulin 924 ng/ml Normal: TSH, T4, TPA, TA	US: large complex mass, heterogeneous, 9.4 × 8 cm, internal vascularity on doppler evaluation, originating from right side, midline extension, R thyroid lobe not clearly seen separately from mass, largest lymph node 1.9 × 0.4 cm in left upper jugular region CT: large R thyroid mass, compression on adjacent structures, highly suggestive of malignancy	Cellular smear, many papillary follicular epithelial cells, nuclear grooves, foamy macrophages, background of blood	TT with selective right dissection	PTC classic variant invading all the margins in background of CLT	N	24/4	pT3b N1b	Yes	N 6
Naciu et al. 2021 [9]	F 47	3	14	Diffuse anterior neck swelling, firm, not tender, skin overlying NR	Normal: TSH, T4, TPA, TA	US neck: enlarged thyroid, thyroid nodules with solid and cystic components, retrosternal expansion CT scan: massive MNG, extend through soma of C2 to thorax 1.2 cm from aortic arch, 14 cm extension, diameter 7.2 cm, compression of trachea causing R deviation, lumen reduction, compressive effects in pharyngeal –laryngeal neck structure, no cervical lymphadenopathy	Cellular smears, fragments, papillary branching, thyrocytes, dischoesive, clear irregular nuclei, intranuclear inclusion	TT	PTC hobnail features in >30% of neoplastic cells, papillary, micropapillary architecture, psammoma bodies and focal necrosis	Strap muscles, peri-esophageal glands	NR	pT4a	Yes	N 36
Baser et al. 2015 [10]	F 40	12	10 × 12	Trilobed anterior neck swelling, well-defined margins, moves with deglutition, extends from midline to posterior triangle on R side of neck, trans- illuminates under light, no cervical lymph nodes	Normal TFT	US: left lobe 1.2 × 3.9 × 1.3 cm with 2 mm hypoechoic nodule. R lobe compressed by large cystic lesion with solid component 2.9 × 2.2 cm inseparable from the gland, query arising from the gland or lymphangioma MRI: R capsulated macro lobulated cystic homogenous mass 11.3 × 10.2 × 7.6 cm, bulge to subcutaneous plane, extends to R paratracheal region. Polypoidal soft tissue component along cyst medial wall arising from R lobe. Tracheal deviation, mild posterior displacement of carotid vessels, no infiltration to adjacent structures	Suggestive of lymphangioma	Right hemi-thyroidectomy with excision of cystic mass	PTC with cystic degeneration	NR	0/0	NR	N	NR
Patil et al. 2012 [11]	F 60	12 y	25 × 15	Multiple cystic swellings extending from thyroid cartilage to mid sternum, moves with deglutition, R side neck sinus with serous discharge	Normal TFT	Neck, chest X-ray: increased soft tissue density in neck extending to upper sternal region, indenting on trachea US: multiple anechoic areas with calcification, internal septae, extending from carotid level to midsternal level. Arising from thyroid tissue with minimal vascularity suggestive of multicystic goiter/lymphangioma CT: multiple fluid cystic lesions in subcutaneous plane of neck and thorax seems arising from thyroid gland only part of left lobe was visible. No inhomogeneous areas within the cyst. Cyst inseparable from thyroid gland. No infiltration of surrounding structures or lymphadenopathy	Suggestive of nodular goiter	TT done along with excision of all cysts and skin around sinus	PTC, sinus tract was free of tumor cells.	NR	0/0	NR	Yes	N NR
Kalllel et al. 2019 [12]	F 45	15 days	11	Febrile, tender, voluminous, fixed swelling occupying anterior cervical region. Inflamed skin surface with permeation nodule	Hb: 8.3 g/dl, WBC: 13,500 cells/ml, CRP: 42 mg/l, Normal TFT	US: voluminous right laterocervical mass CT: voluminous thyroid mass, multi-cystic in the superficial tissues, compressing the trachea and esophagus, pressing vascular axis of neck	Purulent, inflamed material, no cytologic signs of malignancy	Extended TT	PTC	NR	NR	NR	N	NR

A: age in years; CLT: chronic lymphocytic thyroiditis; CP: clinical picture; CRP: C-reactive protein; CT: computed tomography; D: duration in months; EE: extrathyroidal extension; ESR: erythrocyte sedimentation rate; F: female; FNAC: fine needle aspiration cytology; G: gender; Hb: hemoglobin; HP: histopathology; I: immunohistochemistry; LN/+ LN: no. of lymph nodes harvested/number of positive lymph nodes; M: male; m: months; MNG: multinodular goiter; MRI: magnetic resonance imaging; NR: not reported; N: no; PTC: papillary thyroid cancer; R: right; Re/f: recurrence/during follow up in months; S: size in cm; TFT: thyroid function test; T: treatment; TT: total thyroidectomy; TNM: tumor, lymph node, metastasis staging; TSH: thyroid stimulating hormone; TPA: thyroid peroxidase antibody; TA: thyroglobulin antibody; US: ultrasound; WBC: white blood count; y: years. Focality: unifocal in the current case, not reported in all other cases. Immunohistochemistry: not done in the current case, not reported in all other cases except Naciu et al. [9] which revealed BRAF-V600E mutation +ve.

As for tumor size and lymph node metastasis, PTC is classified into three categories: microcarcinoma ≤1.5 cm, mostly non-palpable with no spread to surrounding lymph nodes; intrathyroidal category is larger >1.5 cm, but still confined to the thyroid capsule; and, the extrathyroidal type, commonly breaching the thyroid capsule, and spreading mostly to the cervical lymph nodes [7], [21]. Our patient was. of the extrathyroidal type, presenting with a huge mass (9 × 9 × 8 cm) extending beyond the midline together with bilateral palpable cervical lymph nodes. Table 1 shows similar or larger-sized swellings, but none metastasized to the surrounding lymph nodes [10], [11], [12]. Others reported a hobnail PTC (classified as an aggressive form of PTC), presenting as a 14 cm neck swelling, but with no metastasis to surrounding lymph nodes (Table 1) [9].

In terms of imaging, US scan has a superior spatial resolution for the thyroid gland, making it the modality of choice [22]. PTC mostly appears as hypoechoic irregular mass with microcalcifications and intranodular vascularity, absent hypoechoic halo, and taller than wider appearance of nodules [23], [24]. Our US is consistent with such description, revealing a large irregular complex heterogeneous mass with internal vascularity and multiple cervical lymph nodes. As in Table 1, most other published reports comprised tumors of less solid and more cystic components as seen in US [10], [11], [12]. An exception was one report where the US showed an enlarged thyroid gland, nodules with solid and cystic components and retrosternal expansion, but no lymph nodes (Table 1) [9].

Where US findings are positive, it should be followed by FNA. Although FNA is the gold standard for PTC diagnosis, it is less sensitive in diagnosing neck masses of cystic component in comparison with solid masses, with a 50%–67% false negative rate [25], [26]. Microscopic assessment of our patient's FNA revealed cellular smear with many papillary follicular epithelial cells, nuclear grooves and foamy macrophages diagnostic for PTC. Our literature review highlights that FNA was diagnostic for PTC only in one other report, revealing cellular smears, fragments with papillary branching and thyrocytes, clear irregular nuclei and intranuclear inclusions (Table 1) [9]. The Table also shows that all reported cases of cystic PTC were without cytologic signs of malignancy suggesting either of lymphangioma or nodular goiter [10], [11], [12].

Further radiological imaging such as computed tomography (CT) is recommended in case of huge thyroid nodules or cancers as in our case, to assess for compression or invasion of surrounding structures and furthermore, to assess for retrosternal extension and abnormal lymph nodes to aid in planning for the accurate surgical intervention [22]. Though our patient had compression, there was no invasion of the surrounding structures or retrosternal region (trachea, esophagus, or jugular vein). Table 1 shows that most patients underwent CT in which only one patient had extension to the thorax requiring wider surgical intervention [11].

As regards to the surgical approach, total thyroidectomy is the gold standard for PTC with nodules >1 cm [27]. Large tumors with gross adherence to the surroundings should be resected en bloc in the initial operation. Intraoperatively, initial attempt should always be to dissect and clear the recurrent laryngeal nerves from the cancer whenever possible to preserve their function. All patients with clinical or radiologic lymph nodes involvement should undertake lateral neck dissection with the thyroidectomy and central lymph node dissection is carried out in both involved central lymph nodes and in advanced primary tumors (t3, t4) [28]. Our patient's intervention is consistent with these guidelines, as after discussions at our thyroid MDT, she underwent total thyroidectomy with central and selective right neck dissection. Table 1 reveals that all the other published reports underwent total thyroidectomy and cyst excision but no selective or central neck dissection was undertaken mainly due to uninvolved lymph nodes [9], [11], [12], with the exception of one case, where only right thyroid lobectomy with cyst excision was done [10].

In terms of postoperative course and follow-up, the early postoperative period is a favorable time for the use of radioactive iodine ablation therapy (RAI), an accurate targeted therapy that eradicates and ablates all remnants of thyroid tissue in order to destroy all micro metastases, leading to improved disease-free survival [29]. As per the current American thyroid association (ATA) guidelines, high risk patients should receive RAI after surgery [28]. Our patient was high risk, hence she received high dose RAI after surgery. In our review, two cases had no mention whether postoperative RAI was undertaken or otherwise [10], [12], while the other two cases had RAI after surgery [9], [11].

Regarding follow-up, patients are followed semiannually in the first years after surgery then annually to assess recurrence. Our patient demonstrated optimal wound healing and no locoregional recurrence in the first 6 months after surgery. As seen in Table 1, follow-up was reported in only one publication, where the patient was followed for 3 years with no signs of recurrence [9].

## 8 Conclusion

We present the first case of huge classic PTC with spontaneous subcutaneous rupture causing ischemia, necrosis, and perforation of overlying skin leading to inflammation, presenting with pain and tenderness. The patient was approached systematically, underwent US of the neck followed by FNA which was diagnostic for PTC, after which she she underwent urgent total thyroidectomy with central and selective right side neck dissection and debridement of necrotic skin and subcutaneous tissues. Histopathology revealed a pT3b N1b stage tumor for which she received a high dose of postoperative radioactive iodine ablation. The patient recovered well, with good healing and no recurrence at the 6 month follow-up. Although classic PTC is considered an indolent variant with slow progression, it should be kept in the differential diagnosis of fast-growing swellings of the neck.

## Ethical approval

Approved by Medical Research Center, Hamad Medical Corporation (MRC-04-21-488).

## Sources of funding

Nothing to declare.

## Author contribution

Abdelrahman Abusabeib: Data curation, Writing - review & editing. Walid El Ansari: Conceptualization, Data curation, Investigation, Methodology, Project administration, Writing- original draft, Writing - review & editing. Mohamed S. Al Hassan: Data curation, Writing - review & editing. Mahir Petkar: Laboratory data, Writing - review & editing. Sugad Mohamed: Conceptualization, Data curation, Investigation, Writing -review & editing. All authors read and approved the final manuscript.

## Guarantor

Prof Dr. Walid El Ansari.

## Registration of research studies

Research Registry Unique Identifying Number: researchregistry6868.

https://www.researchregistry.com/browse-the-registry#home/registrationdetails/60b46cbb41d06b001e37199f/

## Consent

Written informed consent for publication of the clinical details and/or clinical images was obtained from the patient.

## Provenance and peer review

Not commissioned, externally peer-reviewed.

## Declaration of competing interest

Nothing to declare.
